# Professional Well-Being of Practicing Physicians: The Roles of Autonomy, Competence, and Relatedness

**DOI:** 10.3390/healthcare6010012

**Published:** 2018-02-02

**Authors:** Oksana Babenko

**Affiliations:** Department of Family Medicine, Faculty of Medicine & Dentistry, University of Alberta, 6-10 University Terrace, Edmonton, AB T6G 2T4, Canada; oksana.babenko@ualberta.ca; Tel.: +1-780-248-1729

**Keywords:** basic psychological needs theory, autonomy, competence, relatedness, well-being, life satisfaction, engagement, exhaustion

## Abstract

This study investigated the roles of basic psychological needs—autonomy, competence, and relatedness—in physicians’ professional well-being, specifically satisfaction with professional life, work-related engagement, and exhaustion. Using an online survey, quantitative data were collected from 57 practicing physicians. Overall, 65% of the participants were female; 49% were family medicine (FM) physicians, with the rest of the participants practicing in various non-FM specialties (e.g., internal medicine, pediatrics, surgery); and 47% were in the early-career stage (≤10 years in practice). Multivariate regression analyses indicated that of the three psychological needs, the need for relatedness had the largest unique contributions to physicians’ satisfaction with professional life, work-related engagement, and exhaustion, respectively. The unique contributions of the needs for autonomy and competence were relatively small. These findings extend basic psychological needs theory to the work domain of practicing physicians in an attempt to examine underpinnings of physicians’ professional well-being, a critical component of quality patient care.

## 1. Introduction

A decade ago, Berwick and colleagues [1] introduced the “Triple Aim” to improve population health, enhance patient experience, and reduce health care costs. While not stated explicitly, the achievement of the Triple Aim presupposes that health care providers experience professional well-being in order to provide effective care to their patients. Bodenheimer and Sinsky [2] note that practices working toward the Triple Aim (e.g., electronic health record technology; documentation), while intended to optimize health care, can negatively affect professional well-being of health care providers and lead to a host of costly outcomes. Work-related burnout and dissatisfaction among health care providers were reported to be contributing factors in the overuse of resources, prescription of inappropriate medications, patient safety, high physician turnover, and early retirement, making the already existing shortage of physicians even more pronounced [2,3]. As such, the importance of Bodenheimer and Sinsky’s call [2] to extend the Triple Aim to the Quadruple Aim by including the goal of improving the work life of health care providers cannot be overstated as it is directly linked to effective and safe patient care. 

The purpose of this study was to investigate the unique roles of autonomy, competence, and relatedness—three basic psychological needs [4,5]—in physicians’ professional well-being, specifically satisfaction with professional life, work-related engagement, and exhaustion. Life satisfaction, and by extension professional life satisfaction, is defined as a conscious cognitive judgement of one’s (professional) life on the basis of the individual’s own unique set of criteria [6,7,8]. Work engagement or rather work disengagement is defined as distancing oneself from one’s work in general, work object, and work content [9,10]. Work-related exhaustion is defined as a consequence of intensive physical, affective, and cognitive strain, that is, as a long-term consequence of prolonged exposure to certain job demands [9,10]. Both disengagement and exhaustion are aspects of work-related burnout [9,10].

According to basic psychological needs theory (BPNT) [4,5], three innate human needs—autonomy, competence, and relatedness—must be supported by the environment for individuals to experience optimal development, functioning, and well-being. The need for autonomy is concerned with the extent to which a person feels that her or his goals and actions are self-chosen and self-endorsed. The need for competence is the degree to which a person deems she or he is effective in one’s own actions and can achieve her or his goals. The need for relatedness refers to the extent to which a person feels connected with and valued by others such as family and colleagues. When these psychological needs are continuously supported by the environment, individuals are more likely to initiate and engage effectively in personal and professional activities and experience well-being (see [5] for the most recent review). In contrast, unmet psychological needs can undermine individuals’ functioning and negatively affect their quality of life. 

Consistent findings with respect to the facilitative role of overall need satisfaction have been reported in various domains such as family, school, relationships, work, and leisure [5,11,12]. Studies in organisational research have shown that need satisfaction is positively linked to well-being [13], intrinsic motivation [4], and higher performance [14] in the workplace, and is negatively linked to distress at work [15]. Research examining the needs for autonomy, competence, and relatedness individually has revealed that each need was positively related to employees’ optimal functioning [16] and intrinsic motivation [17]. To date, however, the roles of autonomy, competence, and relatedness in professional well-being of practicing physicians remain largely unaddressed. 

Based on the findings of published studies conducted with general populations and across various domains [5], this study has three hypotheses:

**Hypothesis** **1.** Satisfaction of physicians’ needs for (a) autonomy, (b) competence, and (c) relatedness at work will positively contribute to (i.e., increase) physicians’ professional life satisfaction.

**Hypothesis** **2.** Satisfaction of physicians’ needs for (a) autonomy, (b) competence, and (c) relatedness at work will positively contribute to (i.e., increase) physicians’ work-related engagement.

**Hypothesis** **3.** Satisfaction of physicians’ needs for (a) autonomy, (b) competence, and (c) relatedness at work will negatively contribute to (i.e., decrease) physicians’ work-related exhaustion.

## 2. Materials and Methods

### 2.1. Procedure

This was an observational survey study. Data from physicians in Canada were collected in September–October of 2017. The link to the questionnaire was circulated using professional mailing lists and word of mouth, including announcements at professional events. Ethics approval was obtained from the University of Alberta Research Ethics Board prior to data collection. Informed consent was implied by the overt action of completing the survey after reading the information letter. Participation in the study was voluntary and participants could choose not to respond to a question if they did not feel comfortable. 

### 2.2. Participants

A total of 57 practicing physicians participated in the study; 11 physicians chose not to answer background characteristics questions but filled out the rest of the questionnaire. Of those who answered demographic questions, 65% of the participants were female; 49% were family medicine (FM) physicians, with the rest of the participants being in various non-FM specialties (e.g., internal medicine, pediatrics, and surgery among others); and 47% were in the early-career stage (≤10 years in practice). 

### 2.3. Measures

#### 2.3.1. Basic Psychological Needs

The 12-item Basic Psychological Needs at Work Scale (BPNWS) [18] was used to assess physicians’ psychological needs satisfaction, specifically satisfaction of the needs for autonomy, competence, and relatedness (four items each). Sample items are: “At work, I feel free to execute tasks in my own way” (autonomy; α (Cronbach’s alpha, internal consistency) = 0.70); “I feel competent at work” (competence; α = 0.73); and “When I am with the people from my workplace, I feel understood” (relatedness; α = 0.88). Participants were asked to indicate how they typically felt in relation to their work using a six-point Likert-type scale (1—strongly disagree; 6—strongly agree). Higher average scores on each need measure were indicative of greater satisfaction of the respective need.

#### 2.3.2. Professional Life Satisfaction

The five-item Satisfaction With Life Scale (SWLS) [7,8] was used to assess the overall satisfaction with professional life (for purposes of this study, the word “professional” was added in each item, i.e., “professional life”). Using a seven-point Likert-type scale (1—strongly disagree; 7—strongly agree), participants were asked to indicate the level of agreement with each statement in relation to their professional life. Sample items are: “In most ways, my professional life is close to my ideal” and “If I could restart my professional life, I would change almost nothing” (α = 0.93). Higher average scores on the scale were indicative of greater satisfaction with professional life.

#### 2.3.3. Engagement and Exhaustion

The 16-item Oldenburg Burnout Inventory (OLBI) [9,10], which consists of two scales, was used to measure levels of work-related engagement and (emotional, physical, and cognitive) exhaustion. Using a four-point Likert-type scale (1—strongly disagree; 4—strongly agree), participants were asked to indicate the level of agreement with each statement in relation to their work. Sample items are: “I find my work to be a positive challenge” (engagement; α = 0.70) and “After work, I tend to need more time than in the past to relax and feel better” (exhaustion; α = 0.81). Higher average scores on each scale were indicative of greater levels of work-related engagement and exhaustion, respectively.

### 2.4. Analyses

SPSS 24.0 (IBM Corp., Armonk, NY, USA) was used to analyze the data (to be available at the institutional research data repository www.library.ualberta.ca/research-support/data-management). Means, standard deviations (SDs), ranges, and bivariate correlations were computed for all study variables. Independent samples *t*-tests were used to test mean differences in the study variables (three psychological needs, professional life satisfaction, engagement, and exhaustion) based on participants’ background characteristics; Bonferroni correction was applied to account for multiple comparisons. Assumptions underlying multivariate regression analysis (linear relationships, multivariate normality, multicollinearity) were checked prior to performing regression analyses. Following this, three multivariate regression analyses were performed to examine unique contributions of the three psychological needs (autonomy, competence, and relatedness) in explaining physicians’ professional life satisfaction, engagement, and exhaustion, respectively. Because the three psychological needs are assumed to be of equal importance, the standard multiple regression was performed by entering the three needs simultaneously in regression analyses. Given a small sample size in the present study, a relaxed *p*-value of < 0.10 was considered statistically significant in regression analyses [19]. 

## 3. Results

As shown in Table 1, physicians in this study reported on average moderately high levels of satisfaction of the three psychological needs: autonomy (M (mean) = 5.35; SD = 0.62), competence (M = 5.10; SD = 0.45), and relatedness (M = 4.79; SD = 0.73). Similarly, the levels of professional life satisfaction (M = 5.24; SD = 1.24), engagement (M = 2.86; SD = 0.36), and exhaustion (M = 2.50; SD = 0.43) among the physicians in this study were on average moderately high. No significant mean differences were observed in the study variables based on physicians’ gender, years in practice, and medical specialty (FM vs. non-FM). Bivariate correlations (Table 1) indicated that the need for autonomy was significantly associated only with professional life satisfaction (r (correlation coefficicient) = 0.39; *p* < 0.01) among the physicians in this study. The need for competence was significantly associated with professional life satisfaction (r = 0.41; *p* < 0.01), engagement (r = 0.28; *p* < 0.05), and exhaustion (r = −0.33; *p* < 0.05). The need for relatedness was significantly associated with professional life satisfaction (r = 0.56; *p* < 0.01), engagement (r = 0.59; *p* < 0.01), and exhaustion (r = −0.35; *p* < 0.01).

Results of the regression analyses are shown in Table 2. The first hypothesis in this study was that satisfaction of physicians’ needs for (a) autonomy; (b) competence; and (c) relatedness at work would positively contribute to (i.e., increase) physicians’ professional life satisfaction. Of the three psychological needs, the unique contribution of the need for relatedness was determined to be statistically significant in explaining physicians’ professional life satisfaction (β (standardized regression coefficient) = 0.46; *p* < 0.001). Contrary to the first hypothesis, the unique contributions of the needs for autonomy and competence, while in the expected directions (i.e., positive relationships), were not significant in explaining physicians’ professional life satisfaction (β = 0.13; *p* = 0.315 and β = 0.20; *p* = 0.135, respectively). Collectively, psychological need satisfaction at work explained 35.3% of the variance in professional life satisfaction among the physicians in this study.

The second hypothesis in this study was that satisfaction of physicians’ needs for (a) autonomy; (b) competence; and (c) relatedness at work would positively contribute to (i.e., increase) physicians’ work-related engagement. Of the three psychological needs, the unique contribution of the need for relatedness was determined to be statistically significant in explaining physicians’ engagement (β = 0.57; *p* < 0.001). Contrary to the second hypothesis, the unique contributions of the needs for autonomy and competence were not significant in explaining physicians’ engagement (β = −0.01; *p* = 0.903 and β = 0.10; *p* = 0.452, respectively). Collectively, psychological need satisfaction at work explained 32.3% of the variance in work-related engagement among the physicians in this study.

The third hypothesis in this study was that satisfaction of physicians’ needs for (a) autonomy; (b) competence; and (c) relatedness at work would negatively contribute to (i.e., decrease) physicians’ work-related exhaustion. Of the three psychological needs, the unique contributions of the need for relatedness and competence were determined to be statistically significant in explaining physicians’ exhaustion (β = −0.28; *p* = 0.039 and β = −0.27; *p* = 0.070, respectively). Contrary to the third hypothesis, the unique contribution of the need for autonomy was not significant in explaining physicians’ exhaustion (β = 0.07; *p* = 0.664). Collectively, psychological need satisfaction at work explained 13.1% of the variance in work-related exhaustion among the physicians in this study.

## 4. Discussion

Published literature reports high levels of burnout and dissatisfaction among physicians and other health care providers globally [3]. In a 2014 survey of US physicians, for example, 46% of physicians reported experiencing symptoms of burnout; 68% of family physicians and 73% of general internists indicated that they would not choose the same specialty if they could start their career anew [2]. In the United Kingdom, almost one-third of the physicians had burnout symptoms; similar findings were also reported in studies from Arab countries [3]. These numbers are alarming as the provision of effective and safe patient care heavily relies on health care providers’ well-being.

The results of the present study revealed that of the three psychological needs, the need for relatedness was significant in explaining all three professional well-being outcomes, specifically physicians’ satisfaction with professional life, work-related engagement, and exhaustion. These findings stand in a sharp contrast with some of the results reported in studies examining the roles of psychological needs in individuals’ satisfaction, engagement, and exhaustion across a variety of domains and achievement settings (e.g., work, leisure, school) (see [5] for in-depth review). 

With respect to satisfaction, in a study conducted with creative employees (e.g., artists, lawyers), sales/service, and working class, Walker and Kono [20] found that the need for autonomy had the largest positive effect on work satisfaction, followed by the need for relatedness; the effect of the need for competence on work satisfaction was determined to be non-significant. These findings suggested that when employees experienced greater satisfaction of their needs for autonomy and relatedness in the workplace, they experienced greater work satisfaction. In the same study, these researchers also examined the effect of employees’ psychological needs satisfaction during leisure on their leisure satisfaction [20]. They found that all three psychological needs were significant in explaining employees’ leisure satisfaction, with the need for autonomy producing the largest unique effect. In the present study, the unique contribution of the need for relatedness to physicians’ satisfaction with professional life was the largest and the only significant effect; unique contributions of the needs for autonomy and competence were not significant. With respect to the non-significant effect of the need for autonomy, it is likely attributed to the high degree of autonomy in physician work in Canada. The high level of autonomy satisfaction that was reported by the physicians in this study supports this explanation. With respect to the non-significant effect of the need for competence, it is likely that its unique contribution to physicians’ professional life satisfaction becomes negligible in face of the much more prominent effect of the need for relatedness.

With respect to engagement and exhaustion, a study examining the role of psychological needs satisfaction among medical students at a Canadian university found that the need for competence had the largest significant contributions to students’ engagement and exhaustion, respectively, followed by the need for autonomy [21]. These findings suggested that when students experienced support of their need for competence in the medical program, students reported to be more engaged with and less exhausted from their studies. Interestingly, in the same study, the researchers observed an unexpected positive and significant relationship between relatedness and exhaustion. Considering that students who participated in that study came from the same medical program, it is possible that one may feel related to others through feeling emotionally, physically, and cognitively exhausted. In the present study with practicing physicians and using the same measures as in the study with medical students [21], the unique contributions of autonomy, competence, and relatedness to physicians’ work-related engagement and exhaustion were markedly different. Specifically, of the three needs, the need for relatedness had the largest significant contributions to physicians’ engagement and exhaustion, respectively. That is, when physicians experienced satisfaction of their need for relatedness at work, they reported being more engaged and feeling less emotionally, physically, and cognitively exhausted. In addition, the unique contribution of the need for competence was significant in explaining physicians’ exhaustion, indicating that when physicians experienced support of their need for competence in the workplace, they felt less exhausted due to work demands. This is not surprising because the dynamic practice of medicine, with new guidelines and developments constantly appearing in the medical field, calls for the work environment that is supportive of physicians’ need for competence and lifelong learning. The latter is ultimately linked to the provision of the effective and safe patient care. 

Finally, the need for relatedness, which in this study was determined to be consistently the largest and significant in explaining the three professional well-being outcomes among practicing physicians, speaks to the importance of creating work environments that are supportive of physicians’ need for relatedness. Due to the increased complexity of health care, more and more often physicians provide patient care and work in interdisciplinary health care teams that include physicians in various medical specialties, nurses, and other allied health care providers (e.g., pharmacists, physiotherapists, dieticians, occupational therapists). At the same time, recent studies report paperwork and administrative tasks to be the leading cause of work-related stress and burnout among physicians, indicating that physicians spend more time on non-face-to-face activities (e.g., letters, medication refills, time-consuming data entry, attending to inbox-type text alerts) than with patients [2]. Clearly, such a work environment does not support physicians’ need for relatedness and their professional call to heal people. Future studies, utilizing qualitative methodology (e.g., interviews and focus groups), are needed to determine optimal ways of supporting physicians’ need for relatedness in the workplace and as such, enhancing physicians’ professional well-being, a critical component of quality patient care.

## 5. Limitations

Several important limitations need to be considered. First, due to the correlational nature of the data, direct causality cannot be inferred from the observed relationships. Second, this study used self-reported data, which may pose concerns around social desirability bias. However, participants tended to respond using the full range of response options, yielding evidence against social desirability bias. Finally, the sample size in this study was small, reflecting the reality of survey studies [22,23]. Nevertheless, Tabachnick and Fidell’s recommendation of at least 10 cases per variable [24] meets the minimum requirement for regression analysis in this study. Despite the small sample size, the association effects (correlations and regression coefficients) observed in this study were moderately strong.

## 6. Conclusions

This study has revealed that psychological need satisfaction at work plays an important role in physicians’ professional well-being. Specifically, physicians who experienced greater satisfaction of their need for relatedness at work reported greater satisfaction with their professional life; more positive work engagement; and feeling less emotionally, physically, and cognitively exhausted. As such, the next step is to determine optimal ways to support physicians’ need for relatedness in the workplace for their positive engagement and professional well-being, and ultimately, for the provision of effective patient care.

## Figures and Tables

**Table 1:** Descriptive statistics for the study variables (*n* = 57).

Variables	Observed Range	Mean	SD	1	2	3	4	5	6
1. Autonomy	3.25–6.00	5.35	0.62						
2. Competence	3.75–6.00	5.10	0.45	0.53 **					
3. Relatedness	1.25–6.00	4.79	0.73	0.34 **	0.31 *				
4. Prof. life satisfaction	1.40–7.00	5.24	1.24	0.39 **	0.41 **	0.56 **			
5. Engagement	1.63–3.63	2.86	0.36	0.23	0.27 *	0.59 **	0.60 **		
6. Exhaustion	1.75–3.50	2.50	0.43	−0.18	−0.33 *	−0.35 **	−0.51 **	−0.50 **	

** *p* < 0.01; * *p* < 0.05; SD: standard deviation.

**Table 2:** Results of multivariate regression analyses.

Outcome Variables	Prof. Life Satisfaction *F* (3,53) = 11.12, *R*^2^*adj* = 0.35, *p* < 0.001				Engagement *F* (3,53) = 9.92, *R*^2^*adj* = 0.32, *p* < 0.001				Exhaustion *F* (3,53) = 3.81, *R*^2^*adj* = 0.13, *p* = 0.015			
Explanatory Variables	B	SE(B)	β	*t*	B	SE(B)	β	*t*	B	SE(B)	β	*t*
Autonomy	0.26	0.26	0.13	1.02	0.01	0.08	−0.01	−0.12	0.05	0.10	0.07	0.44
Competence	0.54	0.35	0.20	1.52	0.08	0.10	0.10	0.76	0.26	0.14	−0.27 *	−1.85
Relatedness	0.78	0.20	0.46 ***	3.93	0.28	0.06	0.57 ***	4.79	0.17	0.08	−0.28 **	−2.11

*** *p* < 0.01; ** *p* < 0.05; * *p* < 0.10; B: non-standardized regression coefficient; SE(B): standard error; β: standardized regression coefficient; *t*: value of the t-test used in significance testing in regression analyses.
